# Sustainable plant polyesters as substrates for optical gas sensors

**DOI:** 10.1016/j.mtbio.2020.100083

**Published:** 2020-10-20

**Authors:** R. Rodrigues, S.I.C.J. Palma, V. G. Correia, I. Padrão, J. Pais, M. Banza, C. Alves, J. Deuermeier, C. Martins, H.M.A. Costa, E. Ramou, C. Silva Pereira, A.C.A. Roque

**Affiliations:** a Instituto de Tecnologia Química e Biológica António Xavier, Universidade Nova de Lisboa (ITQB NOVA), Av. da República, 2780-157, Oeiras, Portugal; b UCIBIO, Chemistry Department, School of Science and Technology, NOVA University of Lisbon, 2829-516, Caparica, Portugal; c i3N/CENIMAT, Department of Materials Science, School of Science and Technology, NOVA University of Lisbon and CEMOP/UNINOVA, Campus de Caparica, 2829-516, Caparica, Portugal

**Keywords:** Cork suberin, Potato suberin, Ionic liquids, Liquid crystals, VOC sensors

## Abstract

The fast and non-invasive detection of odors and volatile organic compounds (VOCs) by gas sensors and electronic noses is a growing field of interest, mostly due to a large scope of potential applications. Additional drivers for the expansion of the field include the development of alternative and sustainable sensing materials. The discovery that isolated cross-linked polymeric structures of suberin spontaneously self-assemble as a film inspired us to develop new sensing composite materials consisting of suberin and a liquid crystal (LC). Due to their stimuli-responsive and optically active nature, liquid crystals are interesting probes in gas sensing. Herein, we report the isolation and the chemical characterization of two suberin types (from cork and from potato peels) resorting to analyses of gas chromatography–mass spectrometry (GC-MS), solution nuclear magnetic resonance (NMR), and X-ray photoelectron spectroscopy (XPS). The collected data highlighted their compositional and structural differences. Cork suberin showed a higher proportion of longer aliphatic constituents and is more esterified than potato suberin. Accordingly, when casted it formed films with larger surface irregularities and a higher C/O ratio. When either type of suberin was combined with the liquid crystal 5CB, the ensuing hybrid materials showed distinctive morphological and sensing properties towards a set of 12 VOCs (comprising heptane, hexane, chloroform, toluene, dichlormethane, diethylether, ethyl acetate, acetonitrile, acetone, ethanol, methanol, and acetic acid). The optical responses generated by the materials are reversible and reproducible, showing stability for 3 weeks. The individual VOC-sensing responses of the two hybrid materials are discussed taking as basis the chemistry of each suberin type. A support vector machines (SVM) algorithm based on the features of the optical responses was implemented to assess the VOC identification ability of the materials, revealing that the two distinct suberin-based sensors complement each other, since they selectively identify distinct VOCs or VOC groups. It is expected that such new environmentally-friendly gas sensing materials derived from natural diversity can be combined in arrays to enlarge selectivity and sensing capacity.

## Graphical abstract

**Figure:**
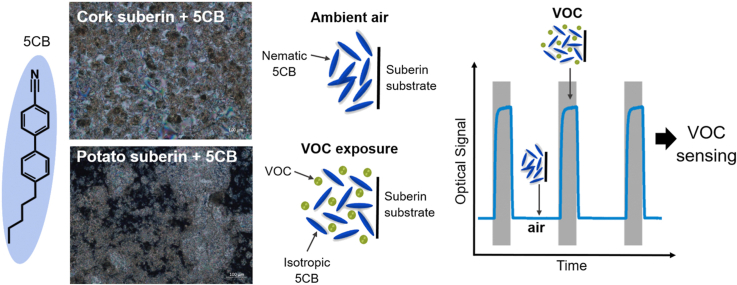

## 1 Introduction

Artificial olfaction using electronic noses is a growing field of interest due to their capability of probing odors and volatile organic compounds (VOCs) in a fast and non-invasive manner. It finds applications in a wide variety of fields, including food and beverage quality control [1], environmental monitoring [2], and diagnostics in healthcare [3,4]. An electronic-nose (e-nose) is a device that reproduces the biological olfactory system, by combining an array of independent partially-specific gas sensors with a signal transduction unit and pattern-recognition algorithms, allowing the differentiation of distinct odors [5]. Gas sensors can be made of different materials. The most common are metal oxide semiconductors (MOS), which require high operating temperatures and power consumption, and synthetic conducting polymers, which have low stability and selectivity. Alternative sustainable materials for gas sensing are emerging, including recent works using bio-based polymers incorporating liquid crystal probes [6] that operate at room temperature with high reproducibility and selectivity [7,8]. Due to their stimuli-responsive and optically active nature, liquid crystals show great potential as optical probes to detect gaseous analytes such as VOCs [6].

Suberin, a complex hydrophobic polyester, is found ubiquitously in land plants, namely in the periderm of tubers, as in *Solanum tuberosum* [9] commonly known as potato, in the endodermis of roots and in the barks of some trees, as in *Quercus suber* [10] commonly known as Cork oaks. It is present in plant cell walls, acting as a protective layer against pathogens and external physical aggressions [11]. Suberin is composed by aliphatic and aromatic monomers, highly cross-linked via ester bonds with glycerol molecules, yielding a macromolecular structure enriched in C20–C24 fatty acids [12,13]. The high content in ω-hydroxyacids and epoxy-fatty acids with mid-chain functionalities have made suberin a polymer of increasing interest useful for the production of novel materials [14,15]. Suberin can be extracted from natural sources through extensive depolymerization using e.g. alkaline hydrolysis or alkaline methanolysis [16]. These unspecific hydrolyses are useful for the determination of the monomeric composition of suberin [17,18], yet its molecular organization is lost. A novel method implementing biocompatible ionic liquids as mild and selective catalysts, results in the extraction of large esterified-suberin structures [13,19], that preserve key native properties, like antibiofouling, antimicrobial activity and moderate hydrophobicity [13,15].

In this work, we report the extraction of the biopolyester suberin from two sources (cork and white potato skin) using the ionic liquid cholinium hexanoate ([N_111_C_2_H_4_OH][O_2_CC_5_H_11_]) to selectively cleave acylglycerol bonds while conserving linear aliphatic ester bonds intact [13,19]. The isolated biopolymers were then used as substrates to immobilize the nematic liquid crystal 4′-Pentyl-4-biphenylcarbonitrile (5CB), and further assessed as potential gas sensing materials for artificial olfaction. Our hypothesis is that the hybrid materials would show distinctive sensing properties because the barrier properties of each suberin type are largely influenced by its structural chemistry. Accordingly, our approach indicates a new unravelled application for plant polyesters-based materials. The possibility to harmonise the tuneable chemical composition of the polyester, defined during the extraction process, with the nature of the analyte to be analyzed, suggests an unprecedented approach towards designed selectivity in environmentally-friendly gas sensing materials.

## 2 Materials and Methods

### 2.1 Chemicals

Cholinium hexanoate was synthesized and characterized as previously described [20]. Hexanoic acid (99.5%) and choline bicarbonate (~80% in H_2_O) were purchased from Sigma-Aldrich (St. Louis, Missouri, USA). The liquid crystal 4-cyano-4pentylbiphenyl (5CB) was obtained from TCI Europe (Zwijndrecht, Belgium). Toluene (pure), dichloromethane (>99%) and ethanol (absolute) were purchased from PanReac AppliCHem (Barcelona, Spain), *n*-hexane (95%) from ThermoFisher (Waltham, Massachusetts, USA) and acetone (100%) from LabChem (Zelienople, Pennsylvania, USA). Acetonitrile (purity ≥99.9%), chloroform, diethyl ether (high performance liquid chromatography [HPLC] grade), ethyl acetate, heptane, methanol were purchased from Fisher Scientific (Hampton, New Hampshire, USA), acetic acid glacial (purity ≥99.7%) and ethanol (≥99.8%) were obtained from Merck, and hexane from VWR (West Chester, Pennsylvania, USA). Solvents were of analytical grade and used as received.

### 2.2 Suberin extraction from plant raw materials

Industrial cork powder (from *Quercus suber* L.) was obtained from Amorim & Irmãos SA (Santa Maria de Lamas, Portugal). This powder, which is chemically similar to natural cork, does not have a proper size for agglomerate production or further uses, hence it is considered an industrial residue. The white potatoes (*Solanum tuberosum* L., cv. Monalisa) were purchased from Batatas Mirense, Lda. (Mira, Portugal) and processed in the laboratory. Potato raw tubers were peeled; the peels were scrapped in boiled water to assure minimum pulp content, and dried at 50 °C until constant weight. Both plant sources were milled (Retsch ZM200 electric grinder; granulometry 0.5 mm; 10,000 rpm) and cleaned from extractives by sequential *Soxhlet* extraction with solvents of increasing polarity (dichloromethane, ethanol and water), as previously described [19]. The extractive-free sources were washed in an excess of deionized water for complete removal of soluble low molecular weight compounds and dried prior to use. Suberin samples were extracted as previously described [20]. Briefly, samples were mixed with cholinium hexanoate for 2 h at 100 °C without agitation. At the end of the reaction, the mixture was filtered (nylon membrane, pore size 0.45 μm, Whatman) to remove insoluble solids. Suberin was recovered from the filtrate through centrifugation (precipitation in an excess of water, 4 °C) and lyophilized.

### 2.3 Suberin chemical characterization

The hydrolyzable monomeric constituents of suberin samples (*ca.* 10 mg) were methylated and trimethylsilylated (hexadecane added as internal standard) prior to quantification by gas chromatography–mass spectrometry (GC-MS) as described before [13]. Briefly, GC-MS (Agilent: 7820 A GC and 5977 B quadrupole MS; HP-5MS column) operated as follows: 80 °C, 4 °C/min until 310 °C; 310 °C during 15 min. Data were acquired using a MSD ChemStation (Agilent); compounds were identified based on EI-MS fragmentation patterns, including the Wiley-NIST reference library and previous published data, and quantified using external standards of the major classes of suberin aliphatic (hexadecanoic acid, hexadecanedioic acid, and pentadecanol) and aromatic monomers (cinnamic acid), at the limits of 5.2–104 μg and 50–1000 μg, respectively. All samples were analyzed in triplicates and also in technical duplicates for a few randomly selected samples. The hydrolyzable samples (aqueous phase) were also used for glycerol quantification by HPLC (Alliance 2695 Waters chromatographer, connected to LKB 2142 Differential Refractometer, with the Empower 2 software, Waters Chromatography) as reported before [13]. Briefly, separation was undertaken at 60 °C using an Aminex HPX-87 column (300 × 7.8 mm), 9 μm particle size (Bio-Rad); elution was carried out isocratically, at a flow rate of 0.5 mL/min; glycerol was quantified using an external calibration curve within the quantification limits of 0.25–9.99 mg/mL.

Nuclear Magnetic Resonance (NMR) spectra were recorded using an Avance II + 800 MHz (Bruker Biospin, Rheinstetten, Germany) spectrometer, as previously described [13]. All suberin NMR spectra (^1^H, HSQC) were acquired in DMSO-*d*_6_ using 5 mm diameter NMR tubes, at 60 °C. MestReNova, Version 11.04–18998 (Mestrelab Research, S.L.) was used to process the raw data acquired in the Bruker spectrometer.

### 2.4 Statistical analyses

The experimental replicas of the relative abundances (mg/g) obtained by GC-MS (Table S1) displayed low variance within each type of suberin, as assessed by the Levene's test: *p-value* = 0.919, *p-value* = 0.907 for potato suberin and cork suberin, respectively. Therefore, the differences of each monomer between the two suberin samples were analyzed using a one-way Analysis of Variance (ANOVA). The contributions of each chemical class and of each monomer to the overall difference between both polymers were calculated using Principal Components Analyses (PCA) upon construction of a Pearson correlation matrix. The analyses were represented in biplots comprising the PCA and the Multidimensional Scaling (MDS) of all contributing points. The statistical analyses were performed using the software XL-STAT v.2014.5.03 (Addinsoft).

### 2.5 Production of films of hybrid materials containing 5CB and suberin

Suberin extracted from cork, and suberin extracted from potato peels were used to produce films of hybrid materials with birefringence and VOC-responsive properties. Briefly, suberin (either from cork or potato peels) was mixed with distilled water (2% w/v), sonicated for 2 h with cycles of 30 min (80 W, 220–240 V) and left for 45 min under agitation (700 rpm) in an 80 °C bath. Afterwards, 5CB was added to the hot mixture (4.6% 5CB v/v) and vortexed for 10 min. Lastly, 5 μl of the final mixture was deposited onto a 20 mm^2^ circular area in an untreated glass slide by drop-casting, and kept for 15 min in an incubator previously heated to 50 °C. To produce films of suberin without 5CB, the same procedure was followed except for the addition of 5CB.

### 2.6 Characterization of the films: surface topography, birefringence and chemistry

The surface topography of the casted materials (with and without 5CB) was observed by Scanning Electron Microscopy (SEM). SEM images were obtained on a Zeiss Auriga CrossBeam workstation equipped with a focused ion beam (FIB) column. The materials were previously coated with 20 μm of AuPd for better conductivity and placed on a carbon-aluminium support. Polarized Optical Microscopy (POM) was used to investigate the distribution of 5CB on the suberin matrix. POM images were taken with crossed (at 90°) polarizers and complemented with bright field (BF) microscopy images, using a Zeiss Axio Observer. Z1/7 microscope equipped with an Axiocam 503 colour camera and operated with ZEN 2.3 software for acquisition and processing of the images. The films surface composition was characterized by X-ray photoelectron spectroscopy (XPS). XPS was performed with a Kratos AXIS Supra spectrometer using a monochromated Al Kα source, running at 225 W. The detailed spectra were recorded with a pass energy of 5 eV. Charge neutralization with an electron flood gun was employed during the measurements, and all spectra were charge corrected to the C–C, C–H of the C 1s emission at 285 eV.

### 2.7 Acquisition of optical signals from hybrid films upon exposure to VOCs

**Fig. 1:**
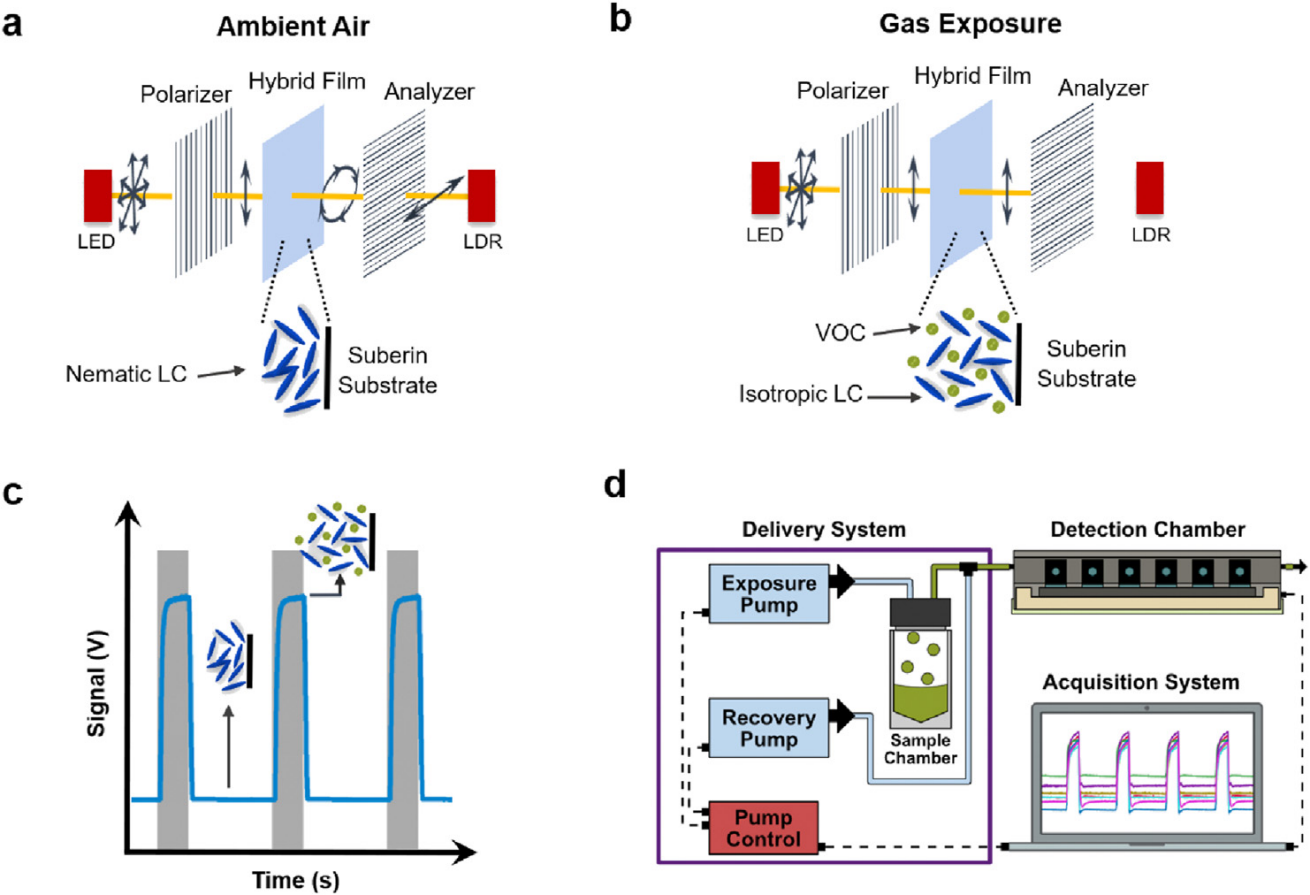
Mechanism of gas sensing using the hybrid films composed of suberin and the liquid crystal 5CB as sensors in an in-house assembled e-nose. **(a, b)** Basic principle of a gas sensor that exploits the birefringence of liquid crystals. **(a)** The sensor is placed between crossed polarizers. At ambient conditions the liquid crystal changes the polarization of the light exiting the light emitting diode, which can be detected by the light receptor (LDR) at the end of the setup. **(b)** when exposed to VOCs, the liquid crystal becomes isotropic and no light reaches the light receptor. **(c)** signal generated by the successive interchange between the nematic and isotropic phase of the liquid crystal within the hybrid suberin films. Gray bars represent gas exposure. **(d)** schematic representation of the in-house assembled e-nose.

Glass slides with drop-casted hybrid films made of suberin and 5CB were used as VOC sensors in an in-house assembled e-nose. The sensing mechanism is based on the birefringence property of 5CB (Fig. 1a–c). Two types of sensors were tested for responses to gas analytes: cork suberin films and potato peels films.

The e-nose device [21] includes a detection chamber, a sample chamber, and an exposure and a recovery pump (Fig. 1d). The detection chamber is connected to the sample chamber containing the VOC sample. The two pumps work alternately. First, the exposure pump drives the vapors from the sample chamber into the detection chamber. Following exposure, the recovery pump pushes ambient air through the system to expel the gas from the detection chamber and attached tubing. The detection chamber, isolated from light and ambient air, includes six independent sensor slots. In each slot a sensor is placed between crossed polarizers (90°) and paired with a light emitting diode (LED) and a light dependent resistor (LDR) (Fig. 1a–b). The output signal for each sensor corresponds to the change in light intensity as detected by the respective LDR during exposure to the gas sample and subsequent recovery with ambient air (Fig. 1c). The analog signal from the LDR is digitalized and stored in a computer for further analysis.

To test the hybrid suberin films for responses to VOCs, the six independent sensor slots of the e-nose detection chamber were occupied with three cork suberin films and three potato peels suberin films. The films were, then, exposed sequentially to the headspace of 12 model solvents, which are structurally similar but from different chemical classes (heptane, hexane, chloroform, toluene, dichloromethane, diethyl ether, ethyl acetate, acetonitrile, acetone, ethanol, methanol and acetic acid), during 21 consecutive cycles. Each cycle comprises 5 s of exposure to the VOC (exposure period) and 15 s of flushing with ambient air (recovery period). The solvents were previously heated to 37 °C for 15 min in a thermostatic water bath. The concentration of VOC in the sensor chamber was calculated between 12 and 15% (v/v), as explained in the detail in a previous publication of the group [8]. Two independent VOC exposure experiments were performed, separated by an interval of 3 weeks, using the same films and the procedure explained above.

### 2.8 Optical signal processing and automatic VOC classification

To process suberin film signals, data analysis tools based on Python libraries (SciPy, sklearn and novanstrumentation) were implemented. The signals were first filtered using the median filter (kernel size equal to 11) from SciPy library, and the smooth function (20 points sliding window) from novainstrumentation library (https://github.com/hgamboa/novainstrumentation). Then, the signals were divided in cycles and the cycles were normalized. In total there were approximately 100 cycles per VOC for each type of suberin film, which were used to train and validate an automatic VOC classifier. Data of cork suberin films was analyzed independently from the data of potato suberin films to allow studying the performance of the two types of sensors individually. For each type of suberin film, the cycles dataset was divided in training dataset (the 50 cycles per VOC from the first experiment) and validation dataset (the 50 cycles per VOC from the second experiment). Twelve features regarding the morphology of the waveform were extracted per cycle, as explained elsewhere [8,21], and used as input to implement automatic VOC classifiers based on the support vector machines (SVM) algorithm. The SVM was tuned with the radial basis kernel and hyperparameters C = 100 and γ = 0.1. The VOC classification results of each type of suberin film were presented in normalized confusion matrices.

## 3 Results and discussion

### 3.1 Extraction and characterization of suberin from cork and potato peels

**Fig. 2:**
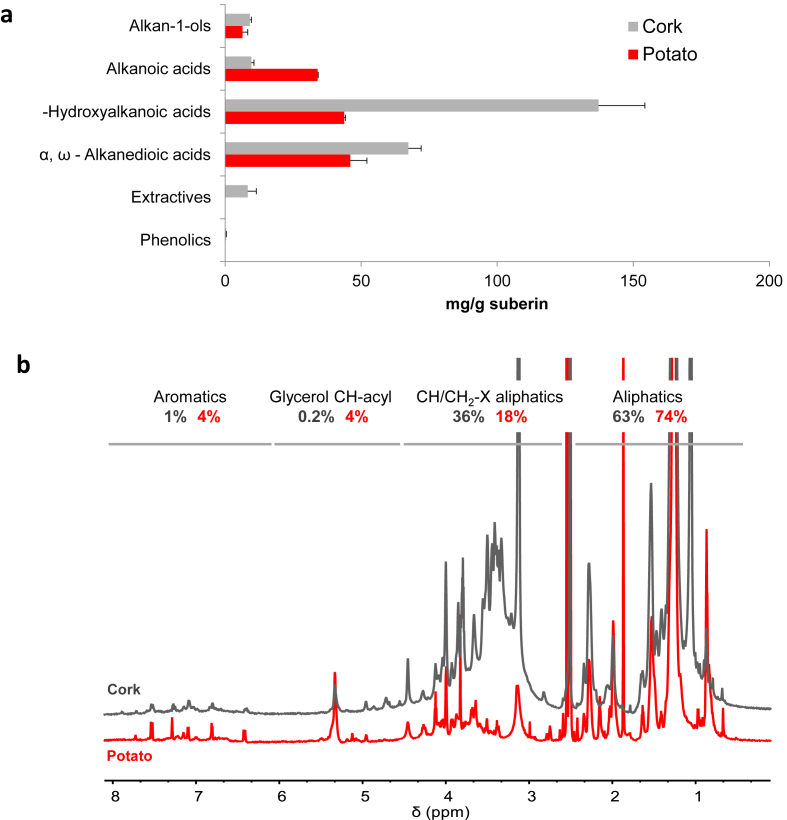
Chemical characterization of cork suberin and potato suberin by GC-MS and wide-ranging NMR analyses. **(a)** Quantitative analysis of the monomeric hydrolyzable constituents of cork suberin and potato suberin by GC-MS. Results are given in percentage as mg of compound *per* g of dried starting material. **(b)** Wide-ranging NMR spectral characterization of the purified suberins. **(b)** The ^1^H NMR with inserts focusing the relative abundance of aliphatics, CH/CH2-X aliphatics, glycerol CH-acyl and aromatics (estimated by integration).

Two sources of suberin, cork and white potato peels, were used to evaluate how differences in the biopolymer structural chemistry could influence the preparation of hybrid films and their VOC-sensing properties. Suberin was extracted from the plant sources via mild cleavage of acylglycerol esters catalysed by cholinium hexanoate [13,19]. This study constitutes the first report on potato suberin obtained using this method. To characterize the ensuing biopolymers, we analyzed their hydrolyzable constituents by GC-MS (Table S1, Fig. 2a) and the whole polymeric molecular structure by solution state NMR (Fig. 2b).

The GC-MS data derived from three different experimental replicas for each suberin type proved to have low variance (Levene's test: *p-value* = 0.919 and 0.907, for potato suberin and cork suberin, respectively). The most abundant monomers were systematically identified in the two suberin types, however the identification yield (estimated through peak area integration) was higher for cork suberin (63.12%) compared to potato suberin (38.18%) (Table S1). The limits on the identification yield by GC-MS are usually attributed to non-volatile high molecular weight oligomeric structures [14]. The most abundant hydrolyzable monomers (accounting for nearly 50%wt) identified in the cork suberin were 22-hydroxydocosanoic acid, 9,10- dihydroxyoctadecanedioic acid and 18-hydroxyoctadec-9-enoic acid, whereas in potato suberin were 18-hydroxyoctadec-9-enoic acid and octadec-9-enedioic acid. In addition, the cork suberin is richer in compounds carrying epoxide and *vic*-diol groups than potato suberin, which has been linked with the formation of relatively strong intra-hydrogen bridges at mid-chain positions [22]. The contribution of each monomer for the separation between the two suberin types was statistically analyzed, reinforcing that their dissimilarity is largely influenced by the most abundant monomers (Principal Components Analyses, Fig. S1).

The monomeric pattern of cork suberin (Table S1) largely matches the previously reported for related samples [13]. For potato suberin, the diversity of monomers and the relative abundance of each class were comparable with those reported before [9], despite differences possibly associated with the extraction method and the raw material. In both biopolymers the more abundant classes of monomers are ω-hydroxyalkanoic acids and α, ω-alkanedioic acids (Fig. 2a, Table S1). Their contribution (mg/g of suberin) to the dissimilarity of the two suberin types was evaluated using Principal Components Analysis computed upon the construction of a Pearson correlation matrix (Fig. S1). The results showed that the dissimilarity is mostly associated with the abundance of ω-hydroxyalkanoic acids in cork suberin, and with that of the α,ω-alkanedioic acids, with a relevant contribution from alkanoic acids, in potato suberin (Fig. S1).

*In planta*, suberin comprises a network of long-chain α,ω-bifunctional acids esterified via glycerol units [13,22]. Therefore, glycerol is an important monomeric constituent of suberin [9,23], as it allows for the formation of a tri-dimensional cross-linked network [24]. Herein, we observed that cork suberin contains twice more hydrolyzable glycerol than potato suberin, namely 4.96% (±0.41) and 2.16% (±0.20), respectively, suggestive of a higher esterification level. The amounts of hydrolyzable glycerol detected in the raw materials were 4.17% (±0.18) and 1.55% (±0.08) for cork and white potato peels (which contained high amounts of starch), respectively. Glycerol levels reported before in cork following removal of non-covalent constituents were *ca.* 4–5% (w/w) [25]. Collectively, these data on the two suberin types support that their polymeric backbones were largely preserved after the ionic liquid extraction.

To gather more information on the polymeric molecular structure of the two suberin types, specifically to their esterification, we resorted to solution state NMR as recently established by us for cork suberin [13]. The NMR characterization of both esterified-suberin structures was performed upon their solubilization in DMSO at 80 °C (Fig. 2b, Fig. S2). The ^1^H and HSQC spectra are comparable to those obtained before for similar samples, and the identification of the ^1^H and ^13^C chemical shifts for the constituent monomers of the two suberin types was based on those previously assigned through a combination of ^1^H–^1^H (COSY) and ^1^H–^13^C (HSQC, HMBC) correlation experiments [13]. The ^1^H spectra of both suberin types and the glycerol CH-Acyl region of HSQC spectra are depicted in Fig. 2b and Fig. S2. respectively. For potato suberin, the relative abundances of aliphatics, CH/CH_2_-X aliphatics, glycerol CH-acyl and aromatics were estimated through the integration of the ^1^H-spectrum as: 74%, 18%, 4% and 4%, respectively. For cork suberin these values were estimated as: 63%, 36%, 0.2% and 1%, consistent to those reported before [13]. Almost no aromatic monomers could be identified by GC-MS (Table S1), contrary to the findings observed in the ^1^H-spectrum (Fig. 2b). This observation verifies our previous claim regarding that both suberin types consist of cross-linked polymeric structures that were not completely hydrolyzed. We also analyzed the relative abundance of each acylglycerol configuration in both suberin type estimated through their contour volume integrals in the HSQC spectra (see full details in Fig. S2) [13]. Both biopolymers contained all the five possible configurations, namely 1,2,3-triacylglycerol (1,2,3-TAG), 1,2-diacylglycerol (1,2-DAG), 1,3-diacylglycerol (1,3-DAG), 2-monoacylglycerol (2-MAG) and 1-monoacylglycerol (1-MAG), as observed before for cork suberin [13], and also in suberin oligomers obtained through a non-specific partial hydrolysis of potato periderms [26]. Collectively, the chemical analyses are suggestive that cork suberin has longer polymer chains and is more esterified and more branched (i.e. higher preservation of aliphatic esters and of glycerol levels, and also higher amounts of TAG and DAG) compared to potato suberin.

**Fig. 3:**
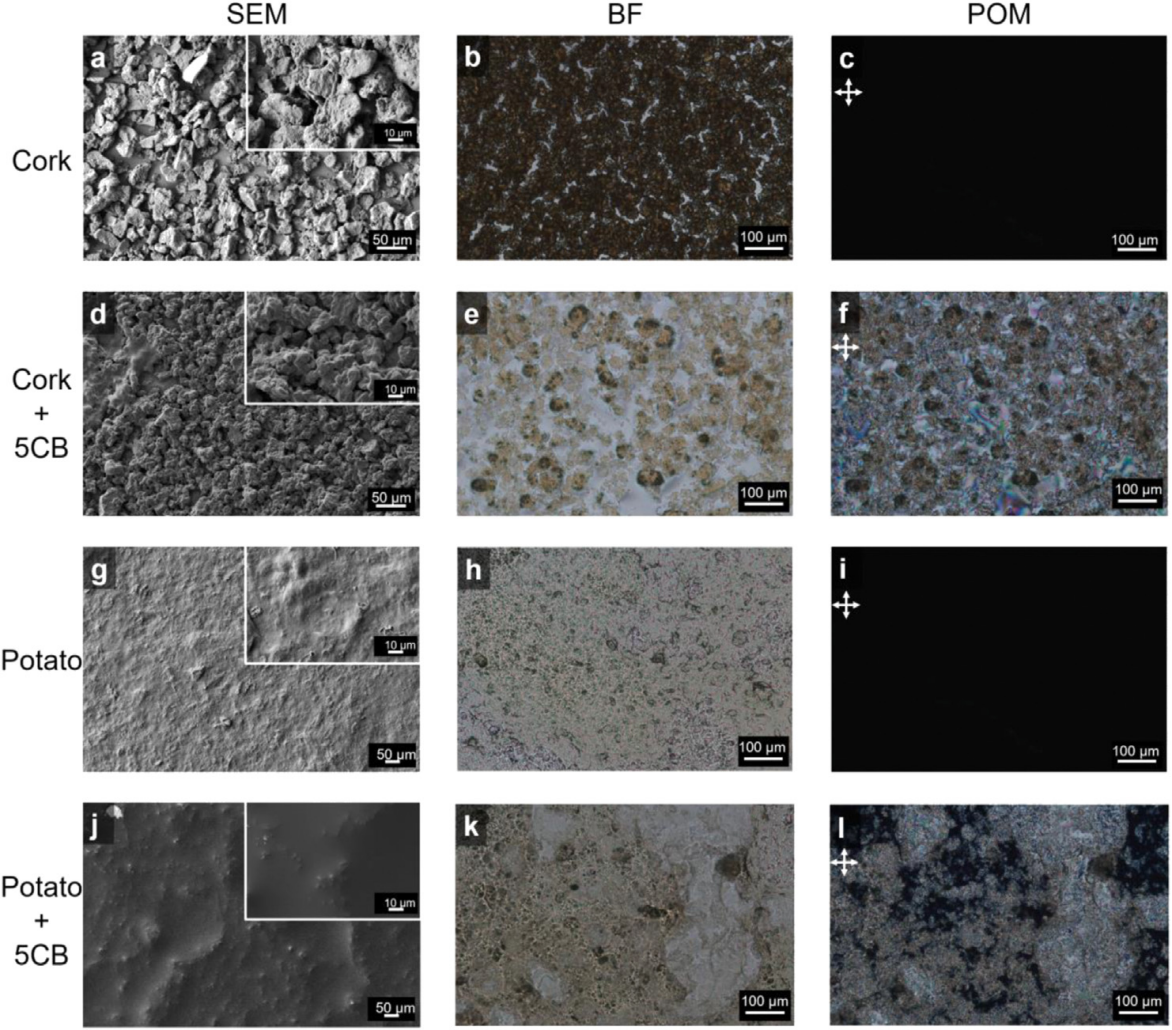
Characterization of cork suberin and potato suberin-based materials regarding morphology and birefringence, using Surface Electron Microscopy (SEM), Bright Field (BF) microscopy and Polarizing Optical Microscopy (POM) with crossed polarizers**. (a**–**c)** Film composed of cork suberin. **(d**–**f)** Hybrid film composed of cork suberin and 5CB **(g**–**i)** Film composed of potato suberin. **(j**–**l)** Hybrid film composed of potato suberin and 5CB. **(a, d, g, j)** SEM images. **(b, e, h, k)** BF images. **(c, f, i, l)** POM images.

Both suberin types were casted onto transparent glass slides, which due to their distinctive polymeric networks rendered very different coating morphologies as observed by SEM imaging. In addition, none of the samples exhibited birefringence under crossed polarizers (Fig. 3c–i). SEM imaging showed that suberin from cork presented a rough surface composed of dispersed and irregular flake-*like* particles of *ca.* 40–50 μm (Fig. 3a), whereas suberin from white potato peel formed a film with a rather smooth surface with small granule-*like* irregularities of <10 μm (Fig. 3g). Previously, using a slow evaporation method, smooth films were obtained with cork suberin displaying fivefold lower esterification level than that obtained here [15]. On the other hand, cork esterified-suberin structures, like those used here, were observed to form large aggregates at a micrometer scale when placed in water, which can assemble into ordered polygonal structures when lyophilized [13]. Hence, the rough topography of cork suberin compared to the smooth one of the potato suberin further reinforces that they comprise large and small cross-linked structures, respectively, as inferred by the NMR data (Fig. 2b and Fig. S2).

In order to fingerprint the surface composition of the two suberin films when casted alone, XPS analyses were undertaken (Fig. S3, Table S2). The atomic concentrations of the more abundant peaks, assigned to C 1s and O 1s regions (nearly 95% of the detected peaks), reveal a higher C/O ratio for cork suberin compared to potato suberin (4.7 vs. 3.8). This is conceivably a consequence of the observed higher proportion of longer aliphatic constituents composing cork suberin as observed by NMR (region of the CH/CH_2_-X aliphatics in Fig. 2b) and also by the GC-MS (Table S1).

### 3.2 Hybrid materials consisting of suberin and 5CB

The basis of LC-mediated VOC sensing lies in the sensitivity of LCs to external stimuli (*e.g.* temperature, electric/magnetic fields or gas analytes) through changes in their molecular order, orientation and/or phase properties. A range of different methods can be used to monitor those changes, often utilizing the birefringence property. A common method is the measurement of the light intensity transmitted through a LC sensor placed between two crossed polarizers (Fig. 1, Fig. 3b). Specific LC orientation patterns, immobilization, and/or confinement of the LC molecules in space, using a variety of system geometries and interfaces, are key features for any LC-based VOC-sensing system. Typical examples are functionalized flat glass surfaces [27], spherical droplets embedded in biopolymer matrices [7,8] and composite fibre mats [28,29].

In this work, the extracted suberin polymers were used as substrates to immobilize the nematic liquid crystal 5CB, based in past work of the group on hybrid sensors [7,8]. The previous hybrid sensors contain gelatin, 5CB and the ionic liquid 1-butyl-3-methylimidazolium dicyanamide ([BMIM][DCA]). In the present work, the relative composition of 5CB was maintained but as suberin was not soluble in [BMIM][DCA], and with choline hexanoate (the ionic liquid used for suberin extraction) no stable materials were obtained, the ionic liquid was not included in the formulation. Suberin and water amounts were set by iterative adjustments until films with immobilized 5CB were obtained.

SEM imaging of both suberin types casted with 5CB in a glass slide, showed that the addition of 5CB resulted in smoother surface topographies (Fig. 3d–j) than those casted alone (Fig. 3a–g). This effect was more pronounced for the cork suberin materials, where the addition of 5CB greatly reduced the surface protrusions, suggestive of ability to disperse the biopolymer particles, possibly through hydrophobic interactions or hydrogen-bonding, via the 5CB cyano moiety.

The hydrophobic suberin polymeric structures are insoluble in water. After adding 5CB and the subsequent drop-casting on the glass surface, water evaporation promotes the sedimentation of suberin particles. 5CB is found mostly on top and between suberin particles, albeit without molecular compartmentalization. This was revealed through POM imaging, as the hybrid materials exhibited birefringence under crossed polarizers (Fig. 3f–l, for cork suberin and potato suberin, respectively), presenting the bright appearance typical of LC molecules oriented in a randomly planar manner on a substrate. 5CB is evenly distributed over the surface of cork suberin as the entire area is birefringent (Fig. 3f). On the contrary, the potato suberin-based hybrid materials exhibited an uneven distribution of 5CB, as POM images present both bright areas (birefringent regions, where 5CB is likely in excess compared to suberin) and dark areas (where suberin is in excess compared to 5CB) (Fig. 3l). POM video recordings of potato suberin materials (Supplementary video) showed that the dark areas stay mostly unchanged during exposure to VOCs and ambient air, thus supporting the hypothesis of low content of the LC in those regions. As the sensing area of the hybrid material is 20 mm^2^, the uneven distribution of 5CB should not affect the response to VOCs.

Supplementary video related to this article can be found at https://doi.org/10.1016/j.mtbio.2020.100083

The following is the supplementary data related to this article:

Video 1 2 Video 1

In the hybrid materials formulation, the liquid crystal is in nearly twofold mass ratio excess relative to the suberin polymer (see Materials and Methods). The surface elemental composition of the hybrid materials consisting of the 5CB with either suberin type was measured by XPS (Fig. S3, Table S2). In both cases, the C/O ratio preserved the trend observed for the biopolymer casted alone, yet increasing to 5.5 and 4.9 for cork suberin and potato suberin, respectively (Table S2). This increase reflects the major contribution of the carbon content of the liquid crystal onto the film's surface. Slight modifications were observed also in the carbon atoms exclusively assigned to suberin (*i.e.* not present in the liquid crystal), some of which support that the biopolymer suffered structural rearrangements when mixed with 5CB. However, the uneven surface distribution of 5CB on the hybrid material of potato suberin, may limit the representativeness of the XPS measurements for these samples.

### 3.3 Analysis of the sensing properties of the hybrid suberin films

The hybrid materials containing 5CB and suberin of either type responded to VOC exposure. It was observed that VOC interaction with the hybrid materials causes a gradual reduction of the order of the 5CB molecules until isotropization. Subsequent exposure to ambient air facilitates the desorption of the VOCs from the film, and consequently the reorganisation of 5CB molecules. These changes in the liquid crystal order generate optical textures when the sample is observed using POM (Supplementary video). They also alter the light intensity transmitted through the film, which can be converted into a measurable signal detected by our custom e-nose [7,8] (Fig. S4).

Similarly to other biopolymer-based hybrid materials reported previously by our group [7,8], the hybrid materials of potato and cork suberin, yielded repeatable and reversible responses to the cyclic exposure and recovery to each of the 12 tested VOCs (variability of 6 ± 2%) (Figs. S4 and S5). Panoramic POM images reveal no significant alteration of the materials’ morphology and 5CB distribution after VOC exposure (Fig. S6), suggesting that both sensor types are stable and resistant to the repeated exposure VOCs at the concentrations used in this work. Three weeks later, after being stored at room conditions, the same sensors were subjected to a second and identical VOC exposure experiment that resulted in response profiles similar to those measured in the first experiment (Fig. S7). Nonetheless, for practical application as re-useable sensors, in the future, longer-term stability will be evaluated.

**Fig. 4:**
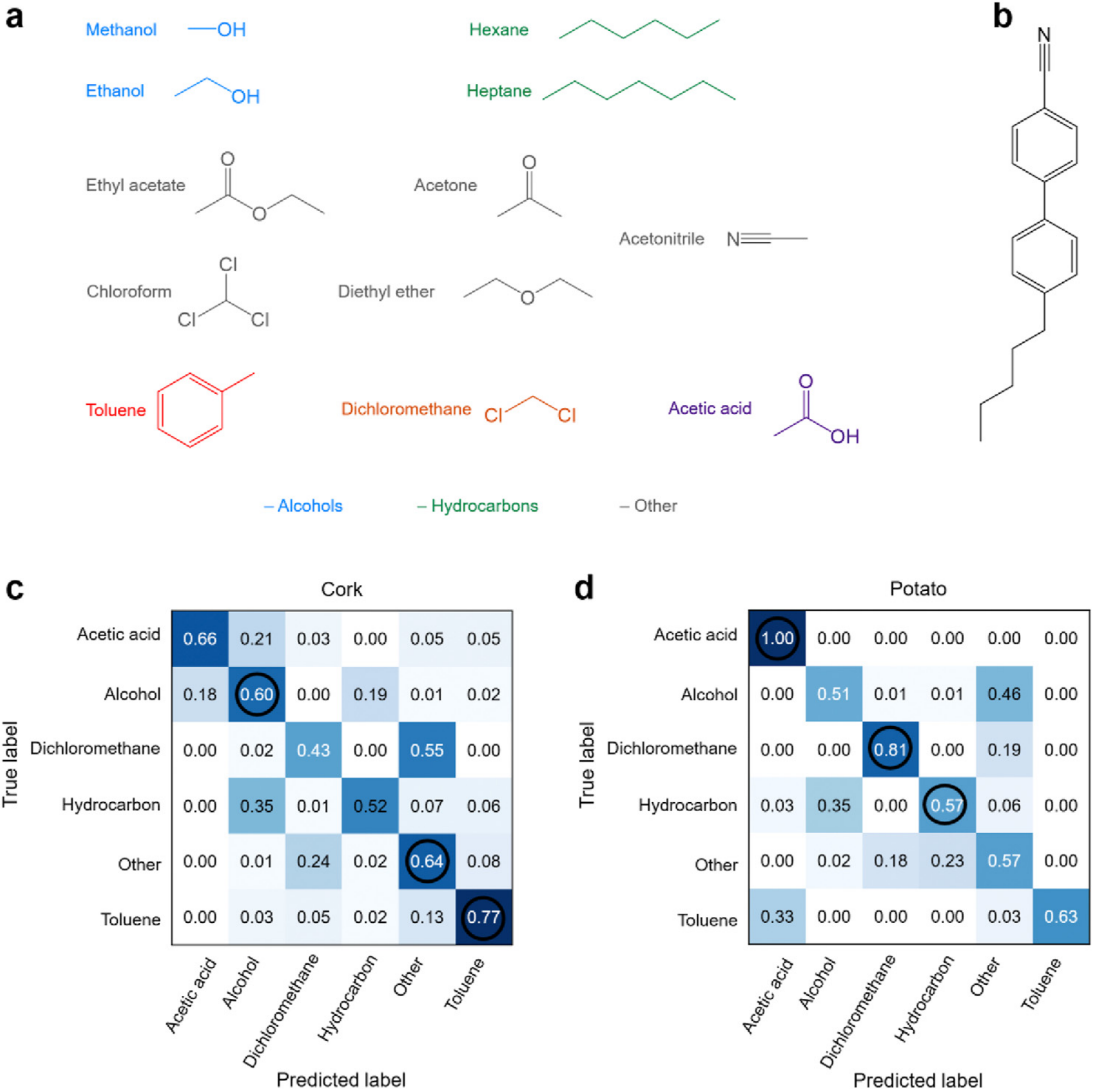
Classification results of support vector machines (SVM) automatic classifiers implemented based on features of the optical signals yielded by suberin-based hybrid films. **(a)** Chemical structures and grouping of the 12 tested VOCs in 6 classes. **(b)** chemical structure of the liquid crystal 5CB. **(c)** normalized confusion matrix representing the performance of the SVM classifier trained and validated with the optical signals from hybrid films of cork suberin and 5CB. **(d)** normalized confusion matrix representing the performance of the SVM classifier trained and validated with the optical signals from hybrid films of potato suberin and 5CB. Note: The values in the confusion matrices represent the relative frequency of the class predictions. The diagonal cells represent the correct predictions made by the classifier and associated prediction accuracy. Incorrect predictions are represented outside the diagonal. Circles in **(d)** indicate the complementarity between the two suberin films for the classification of different groups of VOCs.

The profiles of the signals (Fig. S4) were distinct for some VOCs, predominantly the time and rate required for the reorganisation of the LC. It is important to note that these optical signals are correlated with the affinity exhibited by the sensor constituents towards the tested analytes, as reported previously [7,8]. More specifically, the nature of the interaction between the tested VOCs (structures in Fig. 4a) and the hybrid materials strongly depends on the protic and polar character of the functional groups exposed at the sensor surface. For non-polar VOCs (heptane, hexane, toluene and diethyl-ether, all of which are aprotic), the driving force of the interaction with the hybrid material is likely mediated by 5CB (structure in Fig. 4b), even though certain interactions with the aromatic groups and the non-polar CH_3_ moieties of suberin hydrocarbons could also occur. Similarly, polar yet aprotic VOCs (dichloromethane, ethyl acetate, chloroform, acetone and acetonitrile) may preferentially interact with 5CB, whereas polar and protic VOCs (ethanol, methanol and acetic acid) can interact with the protic hydroxyl groups present in ω-hydroxyalkanoic acids that are abundant in both suberin types (Table S1), or with the cyano moiety of 5CB.

Irrespective of the suberin type, in general, the recovery time of 15 s was insufficient for the signals to return to the initial baseline after exposure to VOCs, indicating that the initial ordering state of the LC is not fully recovered in the used timeframe (Fig. S4). The exceptions to this observation are heptane and hexane, for both types of suberin hybrid material, and ethanol and methanol, for the potato suberin hybrid material (Fig. S4). It is also noticeable that the cyclic exposure of cork suberin hybrid films to ethanol (Fig. S4j) and methanol (Fig. S4k) led to a signal with decreasing amplitude due to the slow recovery of the material to these analytes. We hypothesize that methanol and ethanol strongly interact with the hydroxyl groups and the acylglycerol groups that are more abundant in cork suberin than in potato suberin (Table S1). In addition, the first exposure/recovery cycles of both types of hybrid materials to acetic acid (Fig. S4l) showed an initial increase in the baseline of the signal, which is then maintained through the subsequent cycles. This suggests a possible strong interaction of acetic acid also with the sensing material, possibly forming hydrogen bonds with free hydroxyls groups of suberin or that directly competes with the cyano 5CB moiety to bind to hydrogen bond donor groups in suberin. Any esterification of the free hydroxyls mediated by the acid would be unlikely at the experimental temperature used here.

Since the optical signal waveforms yielded by each type of hybrid material when exposed to the different gas analytes have different features, we hypothesized that the waveform morphology could act as a VOC fingerprint. To test this hypothesis, we utilised machine-learning tools to analyze the optical signal data. The aim was to identify a specific gas analyte through the features of the resulting optical signal. In order to compare the identification (sensing) ability of the two hybrid materials, their optical signals were analyzed independently. First, the complete signals were split into individual cycles, comprised of the combined 5 s exposure time and 15 s recovery time periods. Then, the values of 12 features representing the morphology of the optical signal waveform [8] were calculated for each cycle and used as inputs to develop an automatic VOC classifier based on the support vector machines (SVM) algorithm.

The SVM algorithm was trained with the signals collected in a VOC exposure experiment and validated with the additional data collected in a second experiment (both experiments comprising 50 cycles *per* VOC for each type of hybrid material). This is called independent validation and allows for the evaluation of how the classifier performs when applied to unknown signals. Confusion matrices (Fig. 4c and d, Fig. S8) summarize the classification performance obtained for each hybrid material, specifically the accuracy of the predictions made relative to unknown signals. Contrary to the hybrid materials based on gelatin that we reported previously [8], the suberin-based hybrid materials could not accurately identify individually all the 12 tested VOCs (Fig. S8). This is because certain optical signal waveforms have similar features (Fig. S4) and, thus, do not allow for the distinction of the corresponding VOCs. We then grouped some of the VOCs in classes according to the chemical functionality (alcohols and hydrocarbons) and similarity of the signal waveforms (other), keeping toluene, dichloromethane and acetic acid as individual classes (Fig. 4a) because they were well classified previously (Fig. S5). The classifiers for cork suberin and for potato suberin hybrid materials were then trained and validated with this new VOC class labels. The corresponding confusion matrices (Fig. 4c and d) represent a way of characterizing the sensing ability of the two suberin-based hybrid materials. Cork suberin sensors confused acetic acid mainly with alcohols, but the potato suberin sensors did not confuse acetic acid with any other VOC class. The combination of the two sensors provided a very good distinction of acetic acid, dichloromethane and toluene, with classification accuracies above 70% (Fig. 4c and d). On the other hand, classification of alcohols, hydrocarbons and other VOCs, was less accurate (between 57% and 64%) but still higher than random (50%), confirming the efficacy of SVM training. Remarkably, the two distinct suberin-based sensors complemented each other, since they yield best classification accuracies for distinct VOCs or VOC groups. For example, the potato suberin sensor was more selective towards acetic acid and dichloromethane, while the cork suberin-based was more selective towards toluene. These results push forward the potential use of both suberin-based sensors as two different counterparts of the same gas-sensing array. The observed selectivity towards VOCs typically present in chemical industries production processes (e.g., paper, petrochemical, paints and paint removers) makes such arrays promising for example, towards applications for monitoring product quality or VOC emissions.

Compared to the conventional MOS and conducting polymer-based sensors that usually are used in gas-sensing arrays, the ability of the suberin-based sensors to respond and distinguish high concentrations of VOCs is noteworthy as these conventional gas sensors operate in dynamic ranges only up to ppm concentrations of VOC, losing resolution for higher concentrations, like those tested here, in the range of % vol [30,31].

## 4 Conclusions

Our study presents a new set of gas-responsive films consisting of the plant polymer suberin and the liquid crystal 5CB. Two different raw materials for the extraction of suberin were used: cork and potato peels, rending cross-linked suberin structures of distinctive composition and structural chemistry. These differences have produced 5CB hybrid materials with distinct sensing properties when exposed to different VOCs in the same range of concentrations (12–15% v/v). The optical responses generated by the sensing materials are reversible and reproducible, showing stability upon storage at room conditions for three weeks. Future studies on the sensing performance (such as the limits of detection, long term stability, effect of concentration) will allow a deeper understanding of these hybrid materials as sensors. Since the structural chemistry of suberin, which is found ubiquitously in plants, is plant species and tissue specific, a tremendous variety of distinct cross-linked suberin structures can be exploited. The employed extraction method can be tuned to further increase the diversity of the cross-linked suberin structures, mainly through control of the degree of esterification. Together this offers unlimited flexibility to produce sensors with distinctive sensitivity, in practice it would be possible to select the sensor according to the VOCs to detect. Finally, since the observed responses of the novel suberin-based sensors to the set of 12 different VOCs tested showed complementarity, the use of arrays of distinct suberin-based sensors is a promising alternative to their usage as key elements for future developments of the chemical sensing field, with potential broad applications, e.g. detection of spoiled fish [1] or infectious microbial agents [3].

## CRediT author statement

Rúben Rodrigues, Susana Palma, Vanessa Correia and Joana Pais: Investigation, validation, writing original draft; Marta Banza: Investigation, validation; Cláudia Alves: Formal analysis; Jonas Deuermeier: Investigation; Inês Padrão, Celso Martins, Henrique Costa and Efthymia Ramou: Writing – review and editing; Cristina Silva Pereira and Ana Roque: Conceptualization, methodology, resources, supervision, Writing – review and editing.

## Declaration of competing interest

The authors declare that they have no known competing financial interests or personal relationships that could have appeared to influence the work reported in this paper.
