# Supplementary material for: Sustainable plant polyesters as substrates for optical gas sensors
Source: Mater Today Bio. 2020 Oct 20;8:100083. doi: 10.1016/j.mtbio.2020.100083 (PMC7691741; doi:10.1016/j.mtbio.2020.100083)
Supplement: Multimedia component 1 [file mmc1.docx]

**Supplementary Info**

**Table S1. Quantitative analysis of the monomeric hydrolysable constituents of cork suberin and potato suberin.** GC-MS was used to quantify the hydrolysable monomers of both suberin samples. Results are given in percentage as mg of compound *per* g of dried starting material. The identification yields are indicated below and represent the ratio between the identified peak area and the total area of peaks in the chromatogram. Direct comparison (one-way ANOVA) of the relative abundances of each monomer in each type of suberin are shown.

| **Compounds X** | **mg_X_/g _Suberin_** | | **one-way ANOVA** | |
| --- | --- | --- | --- | --- |
| ***GC-MS*** | **Cork Suberin** | **Potato Suberin** | **F** | ***p-value*** |
| ***Alkan-1-ols*** | **9.09 ± 0.99** | **6.42 ± 0.19** | **21.1066** | ***0.0101*** |
| hexadecan-1-ol | 0.10 ± 0.02 | 0.92 ± 0.05 | 773.6709 | *< 0.0001* |
| octadecan-1-ol | 1.01 ± 0.20 | 2.20 ± 0.05 | 96.8221 | *0.0006* |
| eicosan-1-ol | 0.49 ± 0.04 | 0.51 ± 0.01 | 0.2189 | 0.6642 |
| docosan-1-ol | 1.65 ± 0.20 | 1.42 ± 0.07 | 3.5997 | 0.1306 |
| tetracosan-1-ol* | 5.83 ± 0.67 | 1.37 ± 0.07 | 131.8302 | *0.0003* |
| ***Alkanoic acids*** | **9.65 ± 0.56** | **34.02 ± 1.9** | **453.7117** | ***< 0.0001*** |
| tetradecanoic acid | 0.37 ± 0.06 | 0.63 ± 0.07 | 25.1716 | *0.0074* |
| hexadecanoic acid | 0.88 ± 0.07 | 9.59 ± 0.54 | 774.9171 | *< 0.0001* |
| 9,12-octadecadienoic acid | 0.20 ± 0.07 | 9.68 ± 0.52 | 989.2712 | *< 0.0001* |
| 9-octadecenoic acid | 0.34 ± 0.02 | 5.15 ± 0.75 | 122.0716 | *0.0004* |
| octadecanoic acid | 0.94 ± 0.19 | 3.63 ± 0.08 | 519.1872 | *< 0.0001* |
| eicosanoic acid | 2.52 ± 0.16 | 3.50 ± 0.13 | 69.1826 | *0.0011* |
| docosanoic acid | 4.41 ± 0.32 | 1.84 ± 0.2 | 140.4907 | *0.0003* |
| **ω -Hydroxyalkanoic acids** | **137.22 ± 17.03** | **43.75 ± 0.53** | **90.2388** | ***0.0007*** |
| 16-hydroxyhexadecanoic acid | 2.18 ± 0.20 | 0.44 ± 0.05 | 214.1787 | *0.0001* |
| 18-hydroxyoctadec-9-enoic acid | 28.71 ± 2.50 | 32.25 ± 0.37 | 5.8710 | 0.0725 |
| 18-hydroxyoctadecanoic acid | 0.27 ± 0.23 |  | 3.9992 | 0.1161 |
| 20-hydroxyeicos-11-enoic acid | 4.91 ± 4.26 |  | 3.9819 | 0.1167 |
| 20-hydroxyeicosanoic acid | 4.16 ± 0.40 | 0.46 ± 0.02 | 259.4944 | *< 0.0001* |
| 22-hydroxydocosanoic acid | 51.78 ± 6.38 | 2.28 ± 0.16 | 180.5757 | *0.0002* |
| 24-hydroxytetracosanoic acid | 12.01 ± 1.51 | 3.11 ± 0.32 | 99.2248 | *0.0006* |
| 8, 18-dihydroxyoctadec-9-enoic acid | 1.47 ± 0.06 |  | 1727.0234 | *< 0.0001* |
| ?, 18-dihydroxyoctadec-9-enoic acid | 3.42 ± 0.27 | 1.79 ± 0.13 | 86.2861 | *0.0007* |
| ?,?,?-trihydroxyoctadec-12-enoic acid | 1.01 ± 0.87 | 1.06 ± 0.07 | 0.0117 | 0.9192 |
| 9,10,18-trihydroxyoctadecanoic acid | 1.06 ± 0.92 |  | 3.9992 | 0.1161 |
| 9, 10- epoxy-18-hydroxyoctadecanoic acid* | 26.22 ± 2.01 | 2.35 ± 0.12 | 420.9235 | *< 0.0001* |
| **α, ω - Alkanedioic acids** | **67.37 ± 4.69** | **45.98 ± 6.18** | **22.7903** | ***0.0088*** |
| hexadecanedioic acid | 3.88 ± 0.31 |  | 472.9714 | *< 0.0001* |
| octadec-9-enedioic acid | 9.07 ± 1.20 | 45.25 ± 6.25 | 96.8672 | *0.0006* |
| octadecanedioic acid |  | 0.73 ± 0.08 | 222.7863 | *0.0001* |
| eicosanedioic acid | 2.30 ± 0.11 |  | 1233.0045 | *< 0.0001* |
| 9,10-dihydroxyoctadecanedioic acid | 39.67 ± 2.93 |  | 548.2849 | *< 0.0001* |
| docosanedioic acid | 10.66 ± 0.52 |  | 1268.2788 | *< 0.0001* |
| 9,10-dihydroxyeicosanedioic acid | 1.79 ± 0.15 |  | 412.8570 | *< 0.0001* |
| ***Phenolics*** | **0.35 ± 0.09** |  | **41.1032** | ***0.0030*** |
| 4-hydroxy-3-methoxybenzoic acid (vanillic acid) | 0.35 ± 0.09 |  | 41.1032 | *0.0030* |
| ***Extractives***** | **8.33 ± 3.11** |  | **21.4919** | ***0.0098*** |
| hexadecahydrocyclopentachrysen-9-ol (betuline) | 7.5 ± 2.78 |  | 21.7930 | *0.0095* |
| hexadecahydrocyclopenta chrysene-3-carboxylic acid (betulinic acid) | 0.83 ± 0.33 |  | 18.7037 | 0.0124 |
| * Quantification influenced due to their coelution.  ** Extractives are not considered suberin monomeric constituents. | | | | |

**Table S2**. Component regions atomic concentration (%) of different suberin films (without and with liquid crystal 5CB) as determined by XPS. Detailed C1s region components and respective assignments as well as C/O atomic ratio also shown.

| **Component regions** | **Cork suberin** | **Cork suberin + 5CB** | **Potato suberin** | **Potato suberin + 5CB** | **Assignments** |
| --- | --- | --- | --- | --- | --- |
| **C 1s** | 80.76 ± 0.36 | 82.90 ± 0.45 | 77.02 ± 0.37 | 80.97 ± 0.41 |  |
|  | 50.19 | 56 | 45.72 | 53.01 | C-C, C-H |
|  | 19.48 | 18.64 | 16.14 | 18.03 | C-OH |
|  | 4.17 | 2.12 | 5.75 | 2.53 | epoxy |
|  | 2.99 | 3.5 | 2.52 | 2.52 | C-O-C=O, HO-C=O |
| **O 1s** | 17.01 ± 0.25 | 15.01 ± 0.28 | 20.11 ± 0.25 | 16.69 ± 0.27 |  |
| **N 1s** | 1.78 ± 0.29 | 1.22 ± 0.37 | 1.79 ± 0.32 | 1.71 ± 0.33 |  |
| **P 2p** | 0.00 ± 0.08 | 0.05 ± 0.13 | 0.29 ± 0.11 | 0.30 ± 0.10 |  |
| **Si 2p** | 0.45 ± 0.13 | 0.82 ± 0.17 | 0.79 ± 0.15 | 0.33 ± 0.16 |  |
| **C/O** | 4.7 | 5.5 | 3.8 | 4.9 |  |

**Figure S1.** Biplot (PCA coupled with MDS) showing the contribution of each **(a)** class of monomers and **(b)** monomer for the separation between the two types of suberin.

**Figure S2** Comparative HSQC glycerol CH-Acyl and CH/CH2-X aliphatics region for both cork (grey) and potato peels (red) suberin. Text inserts in the spectra indicate the relative abundance (%) of the different acylglycerol configurations present within each sample as well as the ratio of aliphatic esters and acylglycerol esters. [1] AcylGEs and LAEs stand for acylglycerol esters and linear aliphatic esters, respectively; TAG, DAG, and MAG stand for triacylglycerol, diacylglycerol, and monoacylglycerol, respectively. In both suberin types, most of the retained glycerol was found in the MAG or DAG configurations, with only 4% and 12% as TAG for cork suberin and for potato suberin, respectively. In cork suberin the total amount of MAG equals that of DAG (nearly 48% each), whereas in potato suberin DAG (56%) account for nearly twofold more than MAG (32%). Specifically, cork suberin contains *per* g: 13.4 mg of 1-MAG (27%), 10.4 mg of 2-MAG (21%), 16.4 mg of 1,3-DAG (33%), 7.4 mg of 1,2-DAG (15%) and only 2.0 mg of TAG (4%); while potato suberin contains *per* g: 6.0 mg of 1-MAG (28%), 0.9 mg of 2-MAG (4%), 8.2 mg of 1,3-DAG (38%), 3.9 mg of 1,2-DAG (18%) and 2.6 mg of TAG (12%). We also estimated the ratio of acyl glycerol esters (AcylGEs) and linear aliphatic esters (LAEs) as 73:27 and 83:17 for cork suberin and for potato suberin, respectively.

**Figure S3** C1s, O1s and N1s XPS regions of suberin films with and without liquid crystal 5CB and respective binding energies (eV): a) potato suberin + 5CB, b) potato suberin, c) cork suberin + 5CB, d) cork suberin. C1s region components are also shown for the main functional groups of suberin: C-C, C-H; C-OH; epoxy; O-C=O. C-NH2 is also shown, although it accounts for a minor contribution.


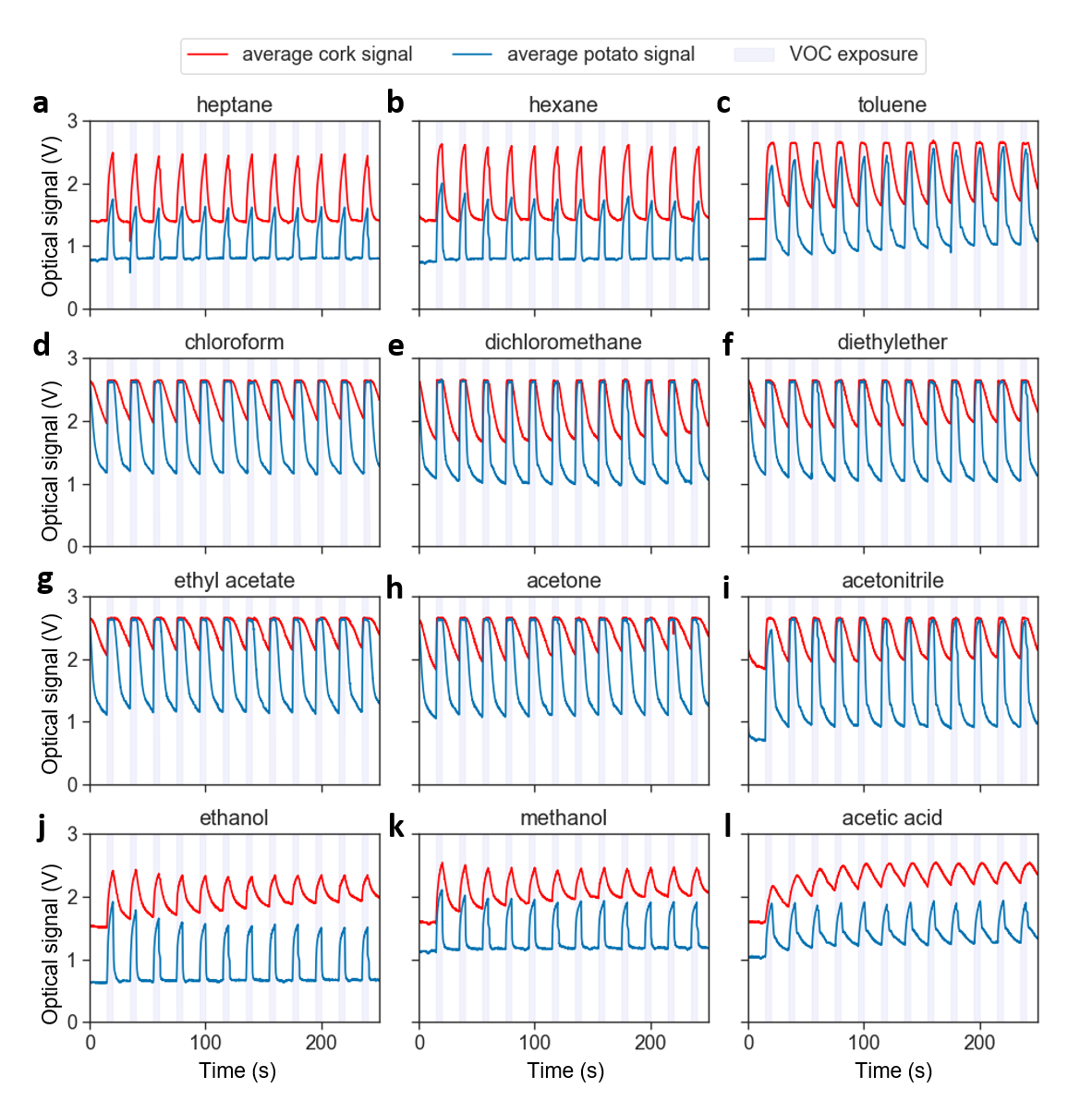


**Figure S4.** Example of the optical signals of cork and potato suberin hybrid films to 12 cycles of exposure (5 s) and recovery (15 s) to 12 different volatile organic compounds (VOCs). The lines represent the average signal of 3 replicate sensors of either type of suberin. VOC exposure periods are highlighted in grey. Recovery periods are in white.


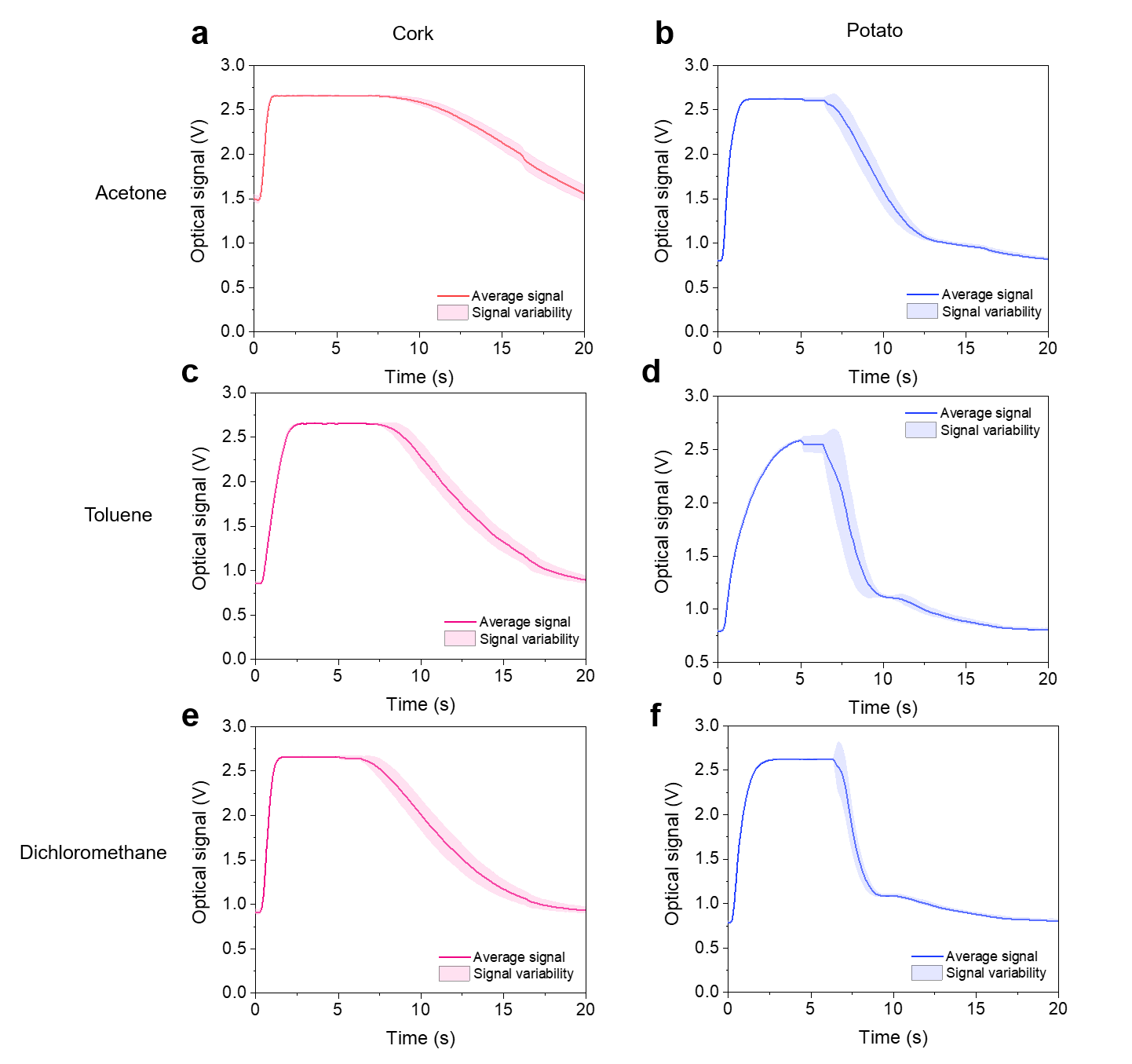


**Figure S5.** Representative examples of the reproducibility of cork and potato suberin-based sensor responses represented as the average and standard deviation of 10 exposure/recovery cycles. (a, c, e) Responses of cork suberin-based sensors; (b, d, f) Responses of potato suberin-based sensors; (a, b) Responses to acetone; (c, d) Responses to toluene; (e, f) Responses to dichloromethane.


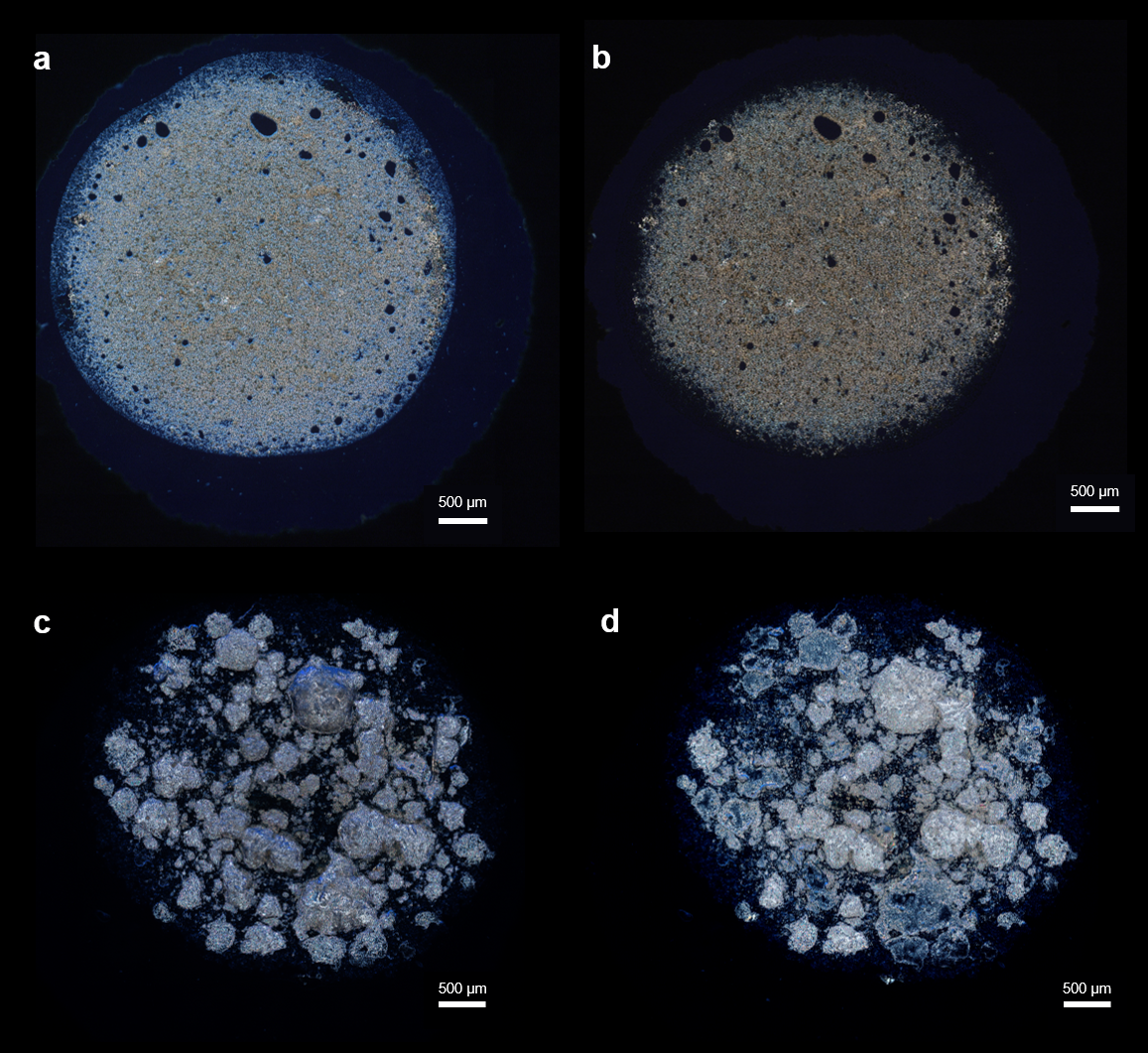


**Figure S6.** Panoramic polarizing optical microscopy (POM) images of the sensing area of cork and potato suberin-based sensors before and three weeks after the first sequential exposure to 12 VOCs. (a, b) Hybrid film sensor composed of cork suberin and 5CB; (c, d) Hybrid film sensor composed of potato suberin and 5CB; (a, c) morphology of the sensing area before VOC exposure; (b,d) morphology of the sensing area 3 weeks after VOC exposure experiment.


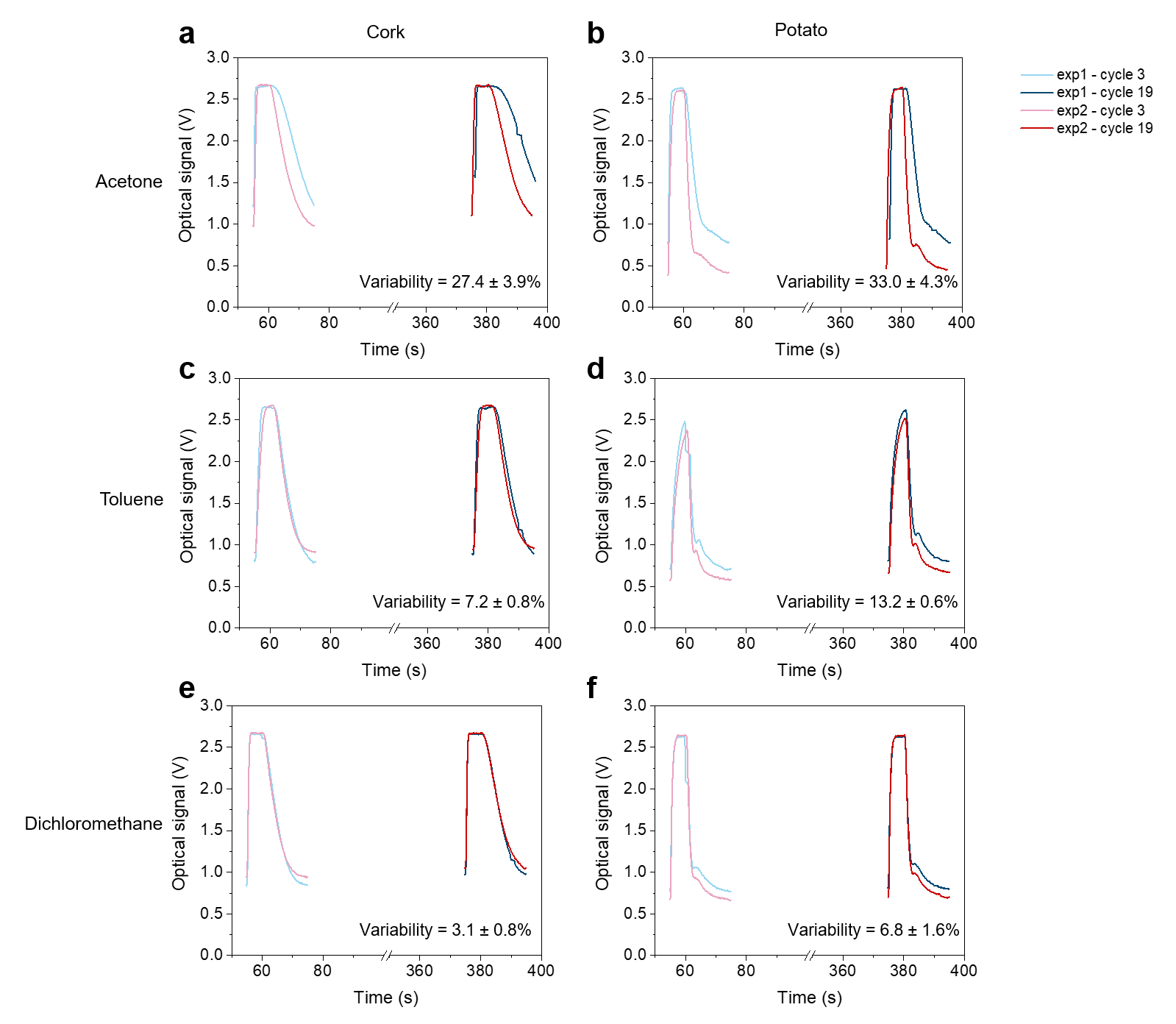


**Figure S7**. Representative examples of the stability of cork and potato suberin-based sensors. The responses to acetone, toluene and dichloromethane are represented for the first experiment and for the second experiment, performed 3 weeks later with the same sensors, stored at room conditions. The responses are identical throughout the 21 cycles of each experiment. Here we represent only the cycles 3 and 19, for clarity. Variability corresponds to the average standard deviation calculated between the responses from the first and the second experiment.


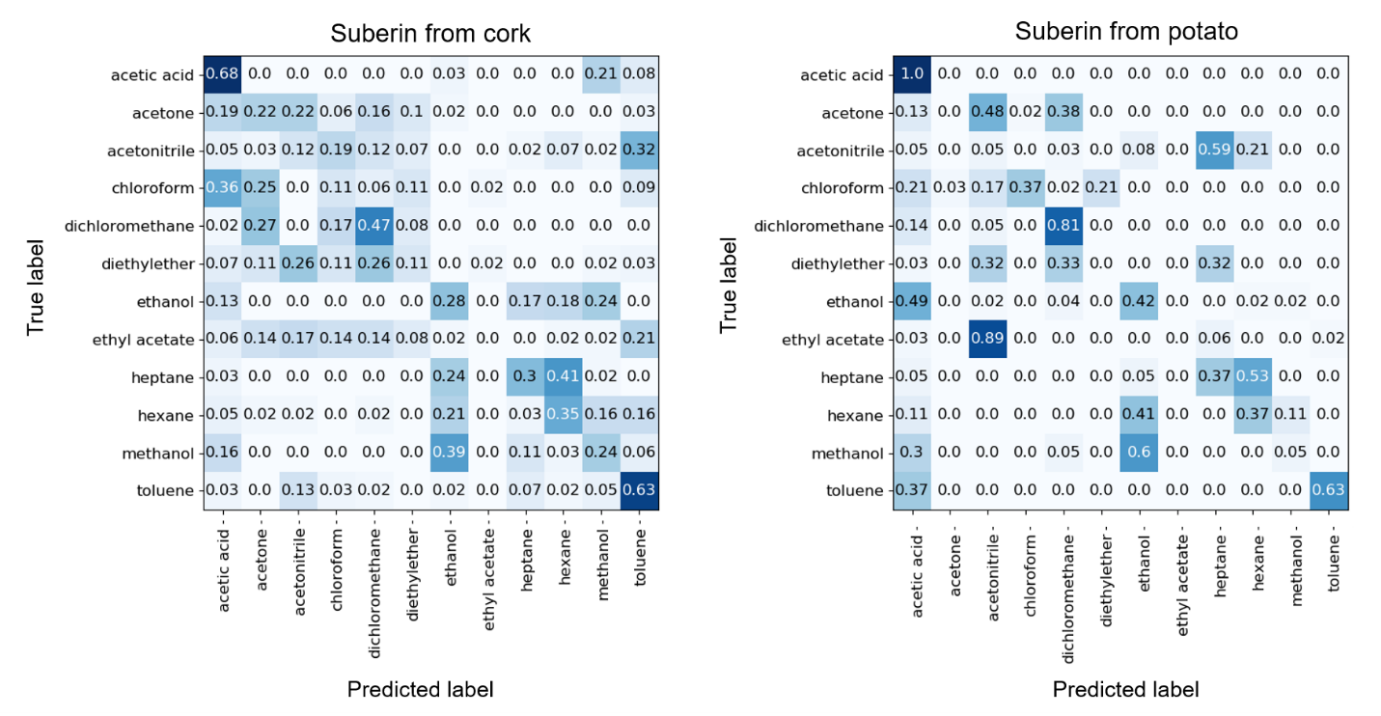


**Figure S8.** Normalised confusion matrices representing the performance of the individual classification of the 12 tested VOCs using the features of optical signals yielded by films made of suberin from cork or suberin from potato combined with 5CB. The values in the confusion matrices represent the relative frequency of each classification. The diagonal cells represent the correct classifications and associated accuracy. Incorrect classifications are represented outside the diagonal. The intensity of blue in each cell represents the performance (increasing performance corresponds to darker blue).

**Supplementary video** – live recording of potato suberin hybrid film responding to dichloromethane gas.

References:

[1] V.G. Correia, A. Bento, J. Pais, R. Rodrigues, P. Haliński, M. Frydrych, A. Greenhalgh, P. Stepnowski, F. Vollrath, A.W.T. King, C.S. Pereira, The molecular structure and multifunctionality of the cryptic plant polymer suberin, Mater. Today Bio. 5 (2020). https://doi.org/10.1016/j.mtbio.2019.100039.
